# Predictive utility of task-related functional connectivity vs. voxel activation

**DOI:** 10.1371/journal.pone.0249947

**Published:** 2021-04-08

**Authors:** Christian Habeck, Qolamreza Razlighi, Yaakov Stern

**Affiliations:** 1 Cognitive Neuroscience Division, Department of Neurology, and Taub Institute for Research on Alzheimer’s Disease and the Aging Brain, and G.H. Sergievsky Center, Columbia University Irving Medical Center, New York, NY, United States of America; 2 Department of Radiology, Weill Cornell Medicine, Brain Health Imaging Institute, New York, NY, United States of America; Boys Town National Research Hospital, UNITED STATES

## Abstract

Functional connectivity, both in resting state and task performance, has steadily increased its share of neuroimaging research effort in the last 1.5 decades. In the current study, we investigated the predictive utility regarding behavioral performance and task information for 240 participants, aged 20–77, for both voxel activation and functional connectivity in 12 cognitive tasks, belonging to 4 cognitive reference domains (Episodic Memory, Fluid Reasoning, Perceptual Speed, and Vocabulary). We also added a model only comprising brain-structure information not specifically acquired during performance of a cognitive task. We used a simple brain-behavioral prediction technique based on Principal Component Analysis (PCA) and regression and studied the utility of both modalities in quasi out-of-sample predictions, using split-sample simulations (= 5-fold Monte Carlo cross validation) with 1,000 iterations for which a regression model predicting a cognitive outcome was estimated in a training sample, with a subsequent assessment of prediction success in a non-overlapping test sample. The sample assignments were identical for functional connectivity, voxel activation, and brain structure, enabling apples-to-apples comparisons of predictive utility. All 3 models that were investigated included the demographic covariates age, gender, and years of education. A minimal reference model using simple linear regression with just these 3 covariates was included for comparison as well and was evaluated with the same resampling scheme as described above. Results of the comparison between voxel activation and functional connectivity were mixed and showed some dependency on cognitive outcome; however, mean differences in predictive utility between voxel activation and functional connectivity were rather small in terms of within-modality variability or predictive success. More notably, only in the case of Fluid Reasoning did concurrent functional neuroimaging provided compelling about cognitive performance beyond structural brain imaging or the minimal reference model.

## 1. Introduction

Functional connectivity has become a major focus of Neuroimaging research in the last 1.5 decades, and the progressive increase in the volume of publications makes it impossible to give an exhaustive literature review. An Endnote search for the keyword “functional connectivity” in the abstract for papers in the 20-year period from 1999 to 2019 yields 22,064 entries, with only 201 entries from 1999–2000, but 6,799 entries from 2017–2018.

While functional connectivity is a data modality that has been researched thoroughly, both as an input and outcome, a simple question concerns the predictive utility of functional connectivity compared to voxel activation, its cousin with a much longer history in neuroscience research. Functional connectivity in our parlance refers to within-subject inter-regional temporal correlation between the signal at locations **x** and **y**, which we will denote as <S(**x**) S(**y**)>. Mean activation at one location, **x**, can be denoted as <S(**x**) d> where *d* denotes a task design vector, which is not dependent on any brain location. (Usually there will be a whole design matrix with multiple columns, but we can forgo this complication for the sake of simplicity.)

The different functional forms for connectivity and activation imply different sensitivities to variation in preprocessing pipelines, and to different kinds of physiological and motion artefacts. This can be appreciated simply from symbolic application of a variational derivative: understanding the dependence functional connectivity on the pre-processing pipeline necessitates the product rule according to

δ<S(x)S(y)>=<(δS(x))S(y)>+<S(x)δS(y)>

while the rule is not necessary for activation since the task design is not dependent on brain location and thus not on pre-processing choice at all. The dependence of functional connectivity on pre-processing and analytic choice, with thorough consideration of motion artifacts in particular, has been discussed at length (see select papers [1–10]), and we cannot possibly do justice to all major contributions so far.

But even under a theoretically possible best processing choice, functional connectivity *might* present as noisier than voxel activation, since functional connectivity is a 2^nd^-order moment, compared to activation (= 1^st^-order) moment, and this could impact the predictive utility of functional connectivity. Since it is difficult to reason about this in the absence of data, an empirical test of brain-behavior predictions with a thorough comparative survey is the best mode of investigation. Brain-behavioral prediction out of sample of course does not capture *all* legitimate research questions answered by neuroimaging analytics. In our crude dichotomy as “input” and “outcome”, it only speaks to “input”, i.e., for predicting as-yet-unknown information from brain, but it is a relevant starting point for connectomics and biomarker research. If mechanistic questions of within-subject regional interactions (=“outcome”) are not of primary concern, the question of relative efficacy of connectomics compared to, and possible synergy with, activation (and indeed brain structure) becomes important. The choice of connectomics as the input for biomarker construction should have empirically verifiable advantages over voxel activation or brain structure regarding predictive utility. Our contention is that, although such comparisons are not overly demanding and quite feasible for any researcher, they have not been undertaken to a sufficient degree.

## 2. Methods

### 2.1 Participant sample

We display some sample demographics of our participants in Table 1 below. All participants were recruited via random market mailing within a 10-mile radius from the hospital location. Informed consent was given according to Columbia University Institutional Review Board guidelines.

**Table 1:** Demographics of the study sample.

**Age: mean ± STD, range**	51.10 ± 16.40, 21–80
**Total number, male, female**	240, 110M, 130 F
**Education in years: mean ± STD, range**	16.24 ± 2.37, 9–24

### 2.2 Data acquisition

All MR images were acquired on a 3.0 Tesla Philips Achieva Magnet. There were two, 2-hour MR imaging sessions to accommodate the 12 fMRI activation tasks as well as the additional imaging modalities, described below. At each session, a scout, T1-weighted image was acquired to determine participant position. Participants underwent a T1-weighted MPRAGE scan to determine brain structure, with a TE/TR of 3/6.5 ms and Flip Angle of 8 degrees, in-plane resolution of 256 x 256, field of view of 25.4 x 25.4 cm, and 165~180 slices in axial direction with slice-thickness/gap of 1/0 mm. All scans used a 240 mm field of view. For the EPI acquisition, the parameters were: TE/TR (ms) 20/2000; Flip Angle 72°; In-plane resolution (voxels) 112 x 112; Slice thickness/gap (mm) 3/0; Slices 41. In addition, MPRAGE, FLAIR, DTI, ASL, and a 7-minute resting BOLD scan were acquired. A neuroradiologist reviewed each participant’s scans. Any significant findings were conveyed to the participant’s primary care physician.

### 2.3 Pre-processing

#### 2.3.1 Structural imaging

Two structural indices were included in our calculations: (1) gray matter volume and (2) thickness, both by region of interest (ROI).

Each participant’s structural T1 scans were reconstructed using FreeSurfer v5.1 (http://surfer.nmr.mgh.harvard.edu/). The accuracy of FreeSurfer’s subcortical segmentation and cortical parcellation [11, 12] has been reported to be comparable to manual labeling. Each participant’s white and gray matter boundaries, as well as gray matter and cerebral-spinal-fluid boundaries, were visually inspected slice by slice, and manual control points were added in the case of any visible discrepancy. Boundary reconstruction was repeated until we reached satisfactory results for every participant. The subcortical structure borders were plotted by *freeview* visualization tools and compared against the actual brain regions. In the case of discrepancy, they were corrected manually. Finally, we computed mean values for 68 cortical ROIs for cortical thickness and cortical volume for each participant to be used in group-level analyses.

These structural variables were combined and used as inputs for our prediction model, similarly to voxel activation and functional connectomes. Cortical volume and thickness are incommensurate, and thus we first z-scored all variables within participant and modality. Subsequently, we concatenated all z-transformed variables to obtain one structural vector with 2*68 = 136 rows. This served as the input to any predictive modeling.

#### 2.3.2 Functional neuroimaging

Each participant’s 12 task-activation fMRI scans were pre-processed in FSL v5.0 [13] using the following steps: (1) within-participant histogram computation for each participant volume to identify noise (FEAT); (2) participant -motion correction (MCFLIRT); (3) slice-timing correction; (4) brain-mask creation from first volume in participant’s fMRI data; (5) high-pass filtering (T = 128 sec); (6) pre-whitening; (7) General-Linear-Model (GLM) estimation with equally temporally filtered regressors and double-gamma hemodynamic response functions; and (8) registration of functional and structural images with subsequent normalization into MNI space (FNIRT).

General linear models (GLM) for each participant and each task consisted of block-based time-series analysis for all tasks. For MEM tasks, in preparation for a prior study [14], we found that that the recognition phase of the trial gives the best correlation with behavior; however, to enable a fair comparison with functional connectivity, we decided to use the whole-task contrasts: restricting the activation only to recognition-phase events would unfairly advantage activation over functional connectivity since connectivity was computed across the whole task block by necessity. Contrary to usual voxel-wise FSL practice, we obtained standardized contrast images of task > fixation for every participant and task to perform group-level multivariate analysis (described in the next section). For all fitted neural responses, incorrect, and correct responses were *not* separated, but analyzed indiscriminately, in the GLM-fitting process.

The voxel size was (3mm)^3^ and we used a probabilistic gray-matter mask to select a subset of voxels with a gray-matter probability P>0.5, resulting in 24,596 voxels being included in the analysis.

#### 2.3.3 Task-related functional connectivity

The slice acquisition delay of Philips scanner was corrected using Sinc interpolation using FSL software package. Well documented motion-induced correlation between ROIs in resting-state and task-based BOLD fMRI (alluded to before) necessitate an extensive correction process. We used mcflirt (motion correction tools in the FSL package [15] to register all the volumes to a reference image [16]. The reference image was generated by registering (6 degrees of freedom, 256 bins mutual information, and Sinc interpolation) all volumes to the middle volume and averaging them. We made sure that the selected middle volume was free of artifacts and motion by examining the derivative of the transformation parameters around that volume. We then used the method described in [17] to calculate frame-wise displacement (FD) from the six motion parameters and root mean square difference (RMSD) of the bold percentage signal in the consecutive volumes for every subject. The contaminated volumes were detected by the criteria FD > 0.5 mm or RMSD > 0.3%. Identified contaminated volumes were replaced with new volumes generated by linear interpolation of adjacent volumes. Volume replacement was done before band-pass filtering [2].

The motion-corrected signals were passed through a band-pass filter with the cut-off frequencies of 0.01 and 0.08 Hz. This band-pass filter has three functions: First, it is an antialiasing filter to remove aliasing due to 0.5 Hz sampling of the BOLD signal; second, it eliminates the higher frequency (>0.1 Hz) fluctuations of the BOLD signal that are mainly a reflection of respiration signal modulated by heartbeat signal; third, it removes the high-power low-frequency noise (the power spectrum of the noise is related to the frequency by 1/f factor). We used flsmaths–bptf to do the filtering in this study [15]. After filtering, the first few volumes were discarded due to the lag of the digital filter. Anecdotal observations in our division showed that digital filter lags (almost the same as the order of the filter) often induce minor correlations between the signals. Finally, we residualized the motion-corrected, scrubbed, and temporally filtered volumes by regressing out the FD, RMSD, left and right hemisphere white matter, and lateral ventricular signals [1].

The residualized time series were then computed for 264 locations in the brain with the taxonomy provided by Power et al. [18]. This resulted in 264 x 264 Fisher-Z correlational matrices which serve as inputs to any group-level analysis discussed in the following. It is possible that some edges are not present because some of the 264 nodes are not all located in gray matter for every subject. We took the conjunction mask of all existing edges across all 240 participants. For 3 references domains, MEM and FLUID all 34,716 edges were utilized; for SPEED and VOCAB only 34,553 and 33,930 edges were used, respectively.

### 2.4 Cognitive tasks

The cognitive tasks used for this study have been described extensively in other publications [14, 19]. We give a short listing of the 12 tasks imaged in the scanner. We covered 4 cognitive domains with 3 tasks per domain: Episodic Memory (= MEM), Fluid Reasoning (= FLUID), Perceptual Speed (= SPEED), and Vocabulary (= VOCAB).

#### 2.4.1 VOCAB tasks

The primary dependent for all VOCAB tasks was the proportion of correct responses.

*2.4.1.1. Synonyms [20].* Participants were instructed to match a given probe word to its synonym or to the word most similar in meaning. The probe word was presented in all capital letters at the top of the screen, and four numbered choices were presented below. Participants indicated which choice was correct.

*2.4.1.2. Antonyms [20].* Participants matched a given word to its antonym, or to the word most different in meaning. The probe word was presented in all capital letters at the top of the screen, and four numbered choices were presented below. Participants indicated which choice was correct.

*2*.*4*.*1*.*3*. *Picture naming*. Participants verbally named pictures, adapted from the picture naming task of the WJ-R Psycho-Educational battery [21, 22].

#### 2.4.2 SPEED tasks

As accuracy for all three SPEED tasks was high, the primary dependent variable was reaction time (RT). For all tasks, participants were instructed to respond as quickly and accurately as possible.

*2*.*4*.*2*.*1*. *Digit symbol*. A code table was presented on the top of the screen, consisting of 9 number (ranging in value from 1–9)-symbol pairs. Below the code table, an individual number/symbol pair was presented. Participants indicated whether the individual pair was the same as that in the code table.

*2.4.2.2. Letter comparison [23].* Two strings of letters, each consisting of three to five letters, were presented alongside one another. Participants indicated whether the letter-strings were the same or different.

*2.4.2.3. Pattern comparison [23].* Two figures, consisting of varying numbers of lines connecting at different angles, were presented alongside one another. Participants indicated whether the figures were the same or different.

#### 2.4.3 FLUID tasks

The primary dependent variable for the Fluid Reasoning tasks was proportion of correct trials.

*2.4.3.1. Paper folding [24].* Participants selected which of 6 options best represented the pattern of holes that would result from a sequence of folds in a piece of paper through which a hole was punched. The sequence was given on the top of the screen, and the six options were given across two rows (three options in each row) below. Response consisted of pressing 1 of 6 buttons corresponding to the chosen solution.

*2.4.3.2. Matrix reasoning (adapted from [25]).* Participants were given a matrix that was divided into nine cells, in which the figure in the bottom right cell was missing. Participants were instructed to evaluate which of eight figure choices, presented below the matrix, would best complete the missing cell.

*2.4.3.3. Letter sets [24].* Participants were presented with five sets of letters, where four out of the five sets had a common rule (i.e. they have no vowels), with one of the sets not following this rule. Participants identified the unique set.

#### 2.4.4 MEM tasks

The primary dependent variable for the MEM tasks was the proportion of correctly answered questions.

*2*.*4*.*4*.*1*. *Logical memory*. Participants were asked to answer detailed multiple-choice questions about a story presented on the computer screen, with four possible answer choices.

*2*.*4*.*4*.*2*. *Word order recognition*. Participants were presented with twelve words sequentially and were instructed to remember the order in which the words were presented. Following the word list, they were given a probe word at the top of the screen, and four additional word choices below. They were instructed to choose out of the four options the word that immediately followed the word given above in the word list.

*2*.*4*.*4*.*3*. *Paired associates*. Participants were instructed to remember pairs of words presented sequentially on the screen. Following presentation of the pairs, participants were given a probe word at the top of the screen and four additional word choices below. Participants were asked to choose the word that was originally paired with the probe word.

### 2.5 Data analysis

#### 2.5.1 Data formats

Both voxel activation and functional connectivity was treated in an identical way by our analytic framework. The input formats are slightly different. Functional connectivity data appears as a 264 x 264 inter-regional Fisher-Z correlation matrix for each 240*12 = 2,880 subjects and tasks. Voxel activation appears as a parametric brain map for each subject and task, resulting in 24,596 (3mm)^3^-voxels that survive a probabilistic gray-matter mask threshold of p>0.5. Both data arrays are shaped into 2-dimensional matrices with as many rows as variables and 2,952 columns. For functional connectivity this implies 264*263/2 = 34,716 rows (the off-diagonal elements in the Fisher-Z matrices), for voxel activation this implies 24,595 rows.

#### 2.5.2 Inter-subject-task variance of voxel activation vs. functional connectivity

Both complete data arrays with 2,880 (= 12*240) observations were subjected to Principal Components Analysis [26, 27], and the grand mean patterns were *not* removed prior to the PCA. Scree plots of normalized Eigen values and cumulative variance spectra were plotted to display the variance concentration and give a sense of the signal-to-noise ratio through the shape of the Eigen value distribution.

#### 2.5.3 Brain-behavioral prediction

Brain-behavioral prediction is the center piece of the current study and serves as the most important metric for comparing the voxel activation and functional connectivity. The prediction framework, Scaled Subprofile Modeling (SSM), has long been established in Neuroimaging analytics [28]. We used a simplified version, which is applicable for both voxel activation and functional connectivity data. Prediction was performed for each cognitive domain separately, and for convenience, cognitive performance, and neural input data (connectivity, activation) were averaged within subject. We performed Monte-Carlo simulation with 5-fold cross-validation, i.e., the data was randomly split into a training set of 192 observations, and a test set of 48 observations repeatedly (= 1,000x). There was no overlap between the participants used in the training and test sets, although there can be repeated measures since every participant has 3 observations for each cognitive reference domain. Prediction performance in the held-out data is quantified with the Predicted Residual Sum of Squares (PRESS) statistic. Line plots for medians and inter-quartile ranges were produced for all 4 cognitive outcomes.

The detailed algorithmic recipe is given here with **1** indicating an intercept term, and ‘indicating matrix transposition. We also included the covariates age, education, and sex in training and test sample, respectively, as **COV**_**1**_ and **COV**_**2**_.

1. Obtain Principal Components, **V**, in training set, **DATA**_**1**_, and fit model to obtain regression coefficients **β** to predict cognitive outcome **Y**_**1**_:
  $$Y_{1}=\left[DATA_{1}\;‘V\left(:,1:N\right)\;COV_{1}\;1\right]\;\beta$$
2. Apply the model to predict the cognitive outcome, **Y**_**2**_, in the test sample **DATA**_**2**_:
  $$pY_{2}=\left[DATA_{2}\;‘V\left(:,1:N\right)\;COV_{2}\;1\right]\;\beta$$
3. Compute the PRESS statistic and compare the predicted to the actual cognitive outcome:
  $$PRESS=<{\left(pY_{2}‐Y_{2}\right)}^{2}>$$
4. Computation of predictive pattern: **pattern** = **V**(:,1:N) **β**(1:N)

The best-fitting PC-set, 1:N, was determined with the AIC criterion [29] in the training set, to avoid over-fitting. We ran the prediction model with the following inputs: voxel activation (ACT), functional connectivity (FC), and brain structure (STRUCT). We also computed the average of all 3 individual-modality predictions (Vote). Lastly, for an honest assessment how much information brain-imaging about the cognitive outcome provides in addition to demographics, we also estimated a reference model using only the covariates age, education, and sex (Reference), i.e., no PCA and pattern derivation form brain-imaging data was performed, and only simple linear regression was used.

Twelve participants (4.88%) had no behavioral data recorded. Their neural data were used in any PCA step, but obviously could not be used for any brain behavioral regression in the training estimation, nor in the out-of-sample testing step.

**Fig 1:**
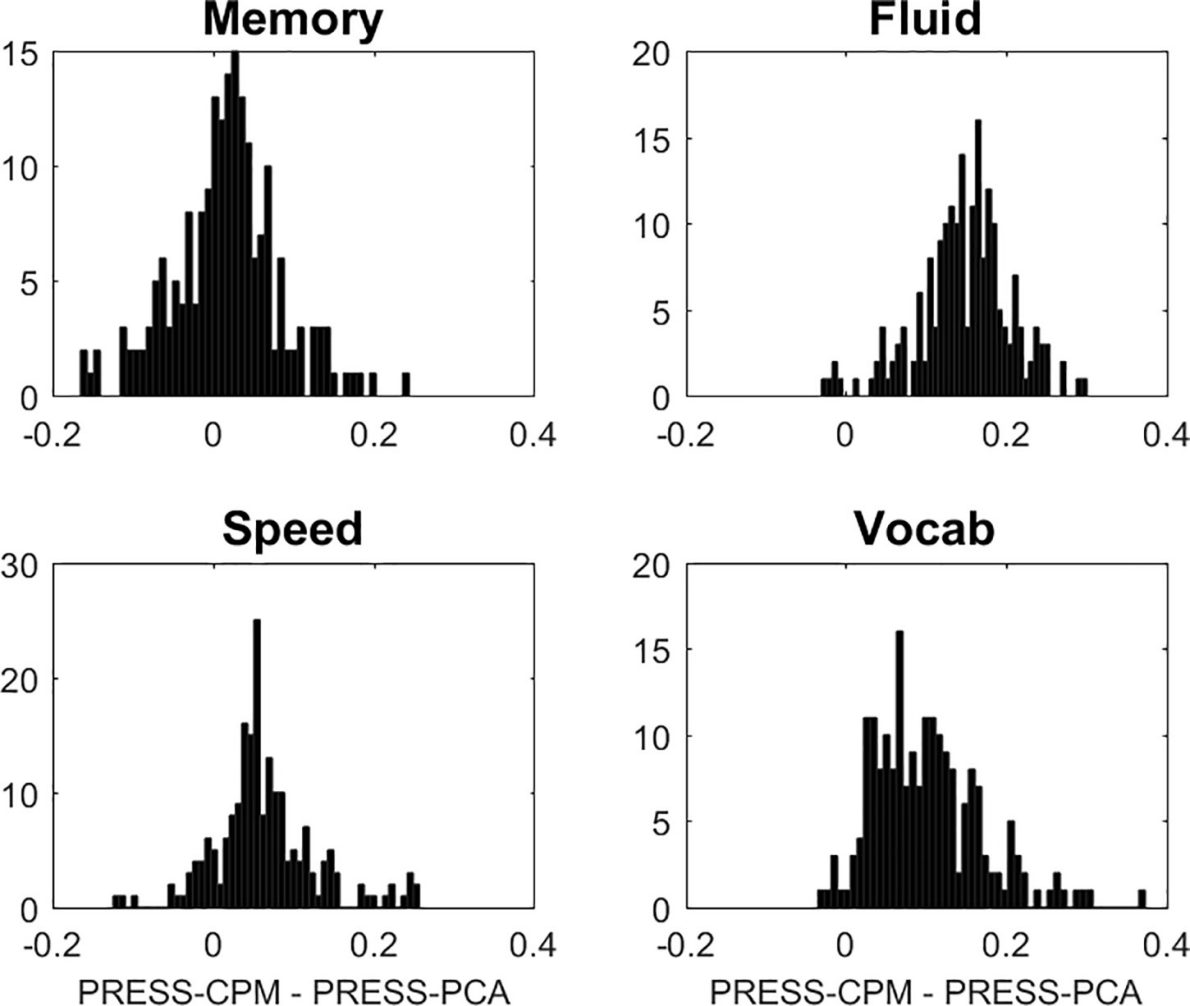
Comparison of CPM to PCA in terms of predictive utility, quantified by PRESS. The mass-univariate p-threshold was 0.05, and we tried more stringent thresholds (p<0.01, p<0.001) too, but the results for CPM were worse. The figure shows histograms of the CPM-PCA differences in PRESS: for all but MEM where CPM and PCA perform roughly equal, PCA performs better than CPM.

For completeness, we decided to include Connectome Predictive Modeling [30] (CPM) in our survey for a brief comparison. CPM is an appealing technique that can serve as a further reference standard for our comparisons. (It is not really restricted to connectivity data per se and could be applied to any high-dimensional data arrays.) Edges that correlate with the to-be-predicted outcome in the training sample are first identified at p<0.05 in mass-univariate screen. A second step in the training sample then involves regressing the outcome against the average value of all such identified edges across subjects. The resulting model can then be fit in the test sample with the quantification of the PRESS-statistic. Since CPM is substantially more demanding because of the mass-univariate pre-screen, we only ran 200 iterations for all cognitive outcomes, and focused only on the comparison between our PCA technique and CPM, leaving out the covariates age, education, and sex, restricting our comparison to the FC-data. In Fig 1 PRESS was shown for all cognitive outcomes to be superior for PCA over CPM. (For completeness we also tried the more restrictive screening criterion and only included edges at p<0.001, but the results came out worse than with the more liberal criterion p<0.05.) Apparently, the summation of edges prior to formulating a regression model loses some of the important information preserved in edge-specific loadings. We stress this is only an isolated comparative assessment of CPM for one data set and one technique; we thus refrained from further discussion of these findings in the paper, and CPM had no further relevance for our paper.

#### 2.5.4 Pattern visualization

1,000 connectivity patterns were derived with a maximum of 34,716 loadings each. We decided to coarse-grain these patterns by average-pooling the between- and within-network connectivity values with the network taxonomy of 14 networks as given by Power et al. [18]. This means that the 264 x 264 connectivity matrix patterns are converted to 14 x 14 matrix patterns. We computed the means and standard deviations across all 1,000 patterns, and computed Z = mean/std for every cell in the 14 x 14 matrix. We thresholded the matrices at |Z|>3.

We also derived summary voxel-activation patterns from 1,000 computed patterns for each cognitive outcome. Z-patterns were computed similarly to the connectivity patterns according to Z = mean/std for all voxel loadings, but no coarse-graining was performed, and voxel-wise patterns were computed and thresholded at |Z|>3.

## 3. Results

### 3.1 Variance spectra

**Fig 2:**
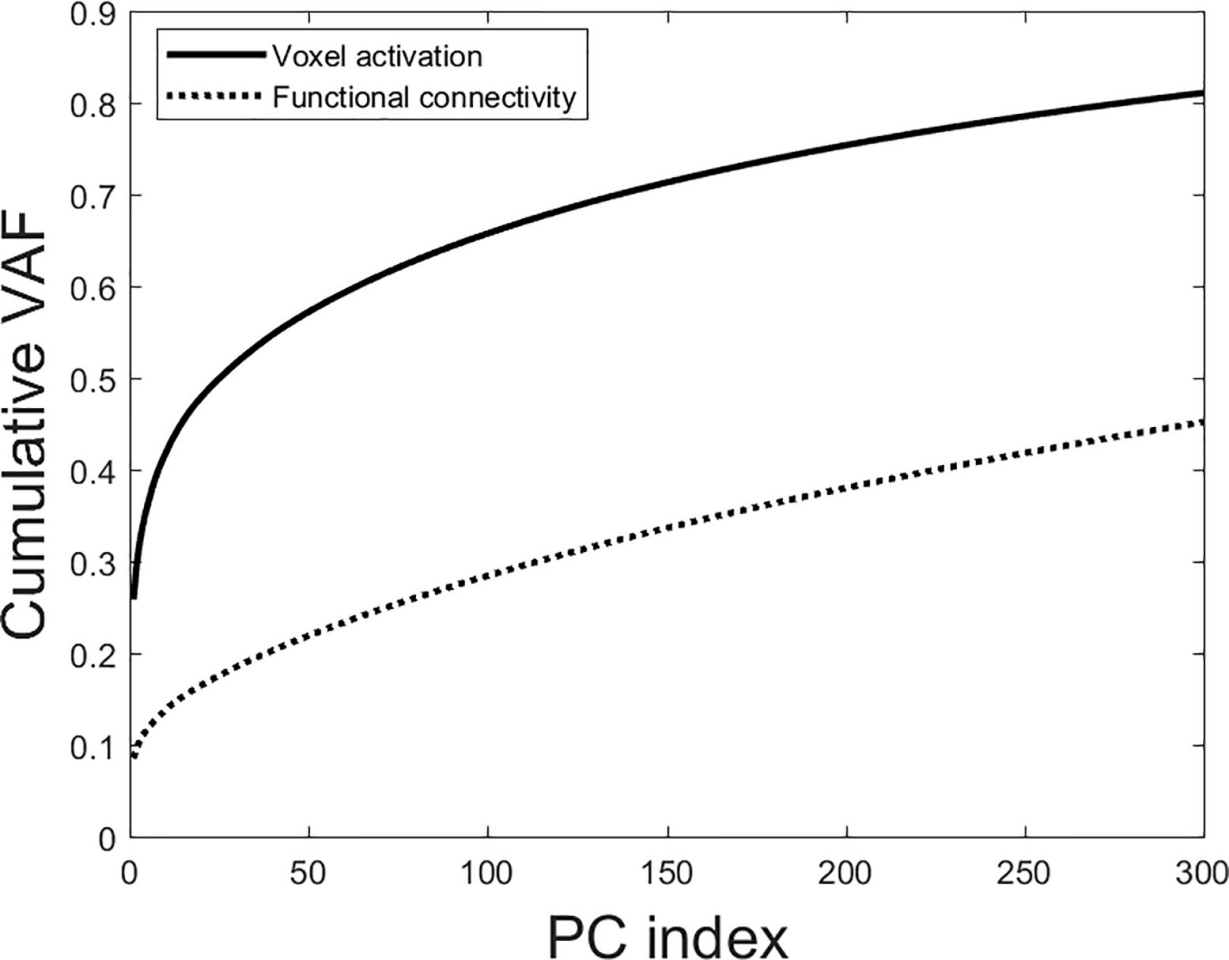
Cumulative scree plots for both voxel activation and functional connectivity for the first 300 PCs. Voxel activation has much better variance concentration, implying better signal-to-noise ratios.

We performed PCA on the total data arrays for both modalities. The cumulative variance spectra are shown in Fig 2.

The cumulative variance spectrum shows voxel activation to concentrate almost double the aggregate variance compared to functional connectivity in the first 300 PCs. Functional connectivity thus appears to be considerably noisier than voxel activation, with a more degenerate variance spectrum.

### 3.2 Brain-behavioral predictions

**Fig 3:**
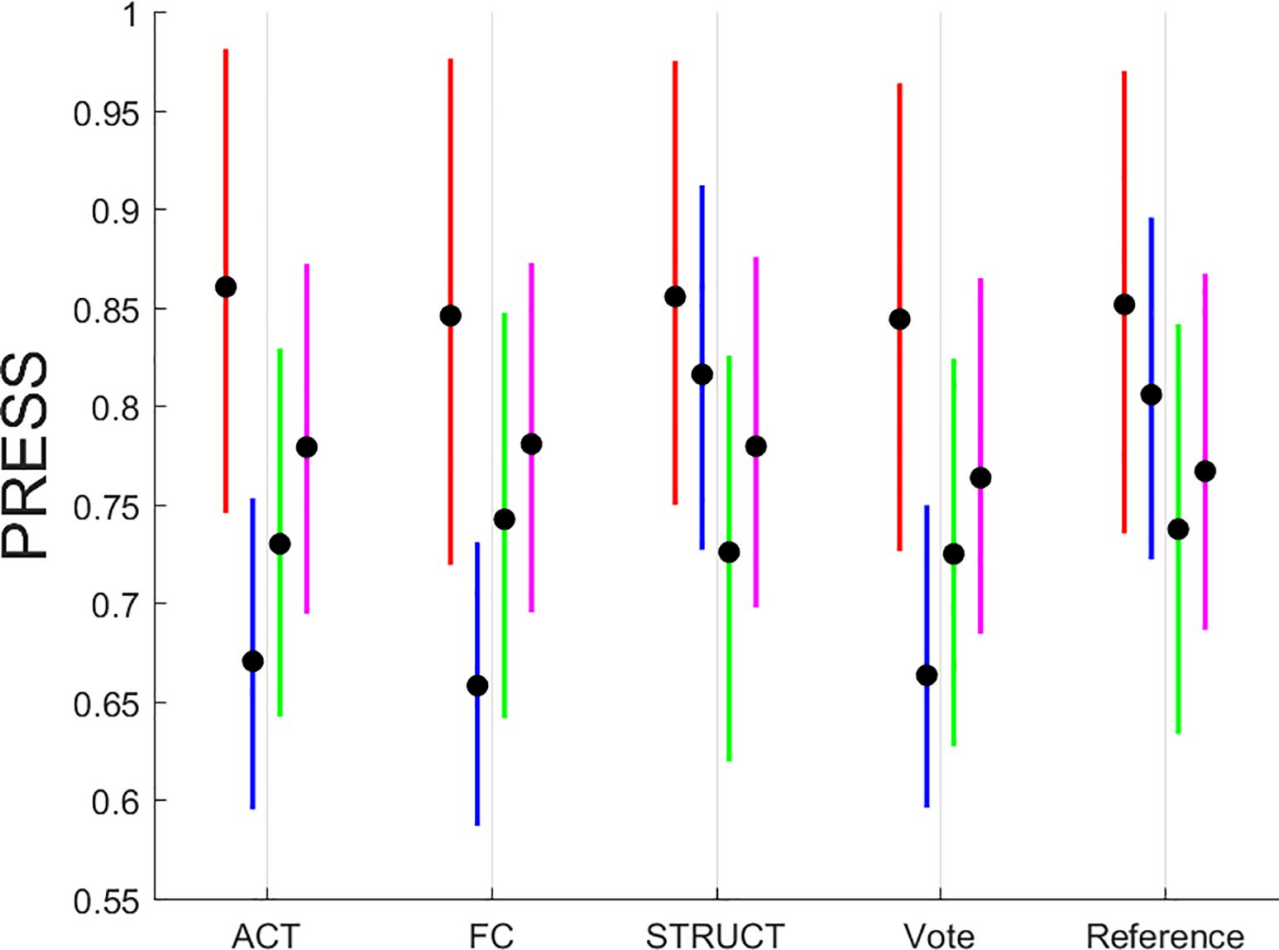
Summary plot of PRESS statistic for all simulations run with 1,000 iterations each. The color scheme is as follows: MEM—red, FLUID—blue, SPEED—green, VOCAB—magenta. Line plots are given for inter-quartile ranges with the median marked with a thick black dot.

A summary display of the PRESS results is shown in Fig 3.

The summary display shows a few salient points: (1) functional connectivity offers modest benefits over activation only for FLUID and VOCAB tasks; (2) structural brain data offer no discernible advantage over functional neuroimaging or reference demographics which affirms that functional neuroimaging during performance of a cognitive task, but, with exceptions of the FLUID tasks, the information provided by structural data is also not appreciably worse than functional brain imaging; (3) brain imaging, both functional and structural, as captured in the “Vote” prediction offers added benefits over the minimal “Reference” model of demographics only for FLUID tasks, with virtually no benefit for MEM, SPEED and VOCAB.

**Fig 4:**
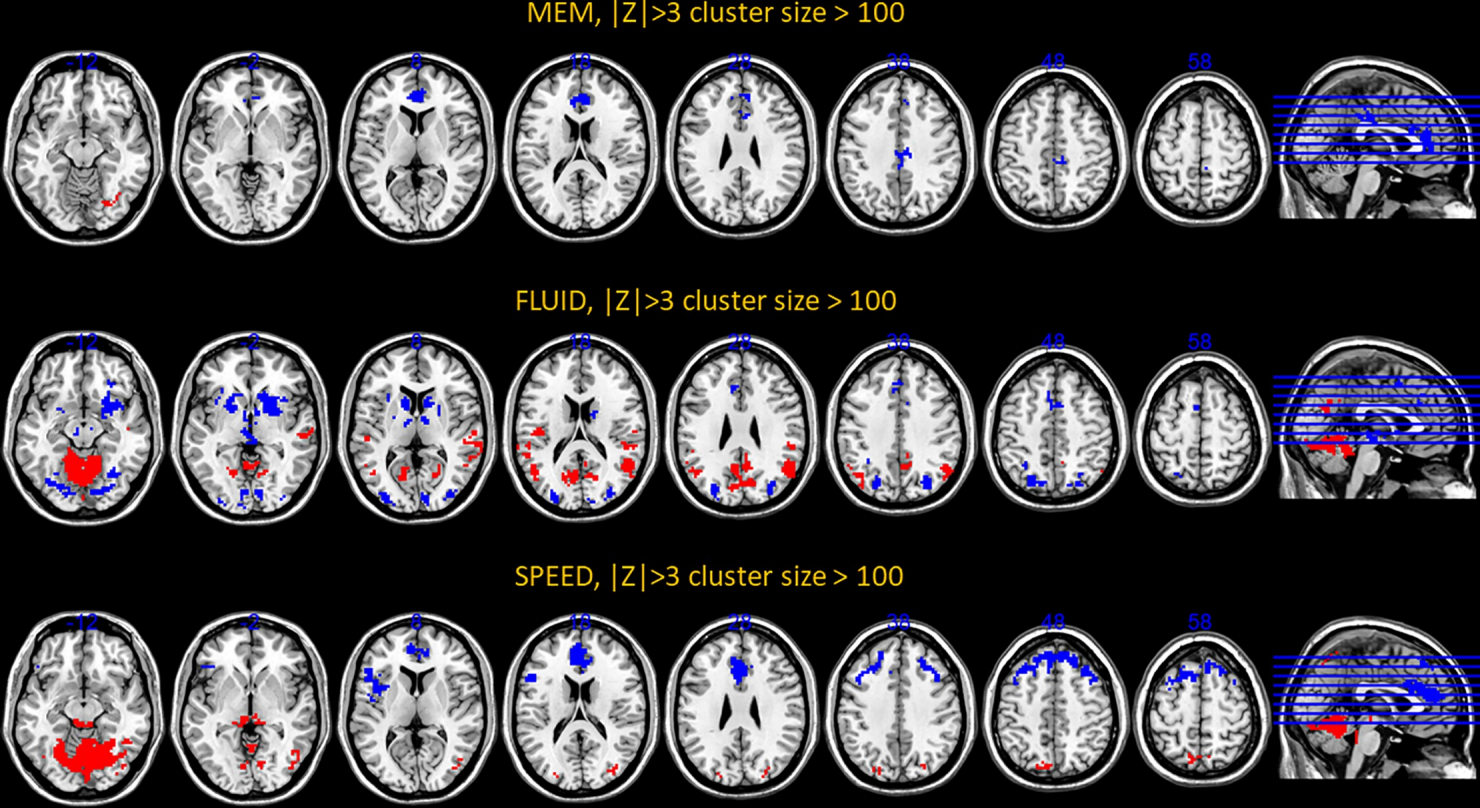
Voxel-activation patterns for all cognitive outcomes but vocabulary positive for which no super-threshold voxels could be located. Positive loadings, Z>3, are denoted with warm colors, while negative loadings, Z<-3, are denoted with cold colors. The cluster-size threshold was 100.

**Fig 5:**
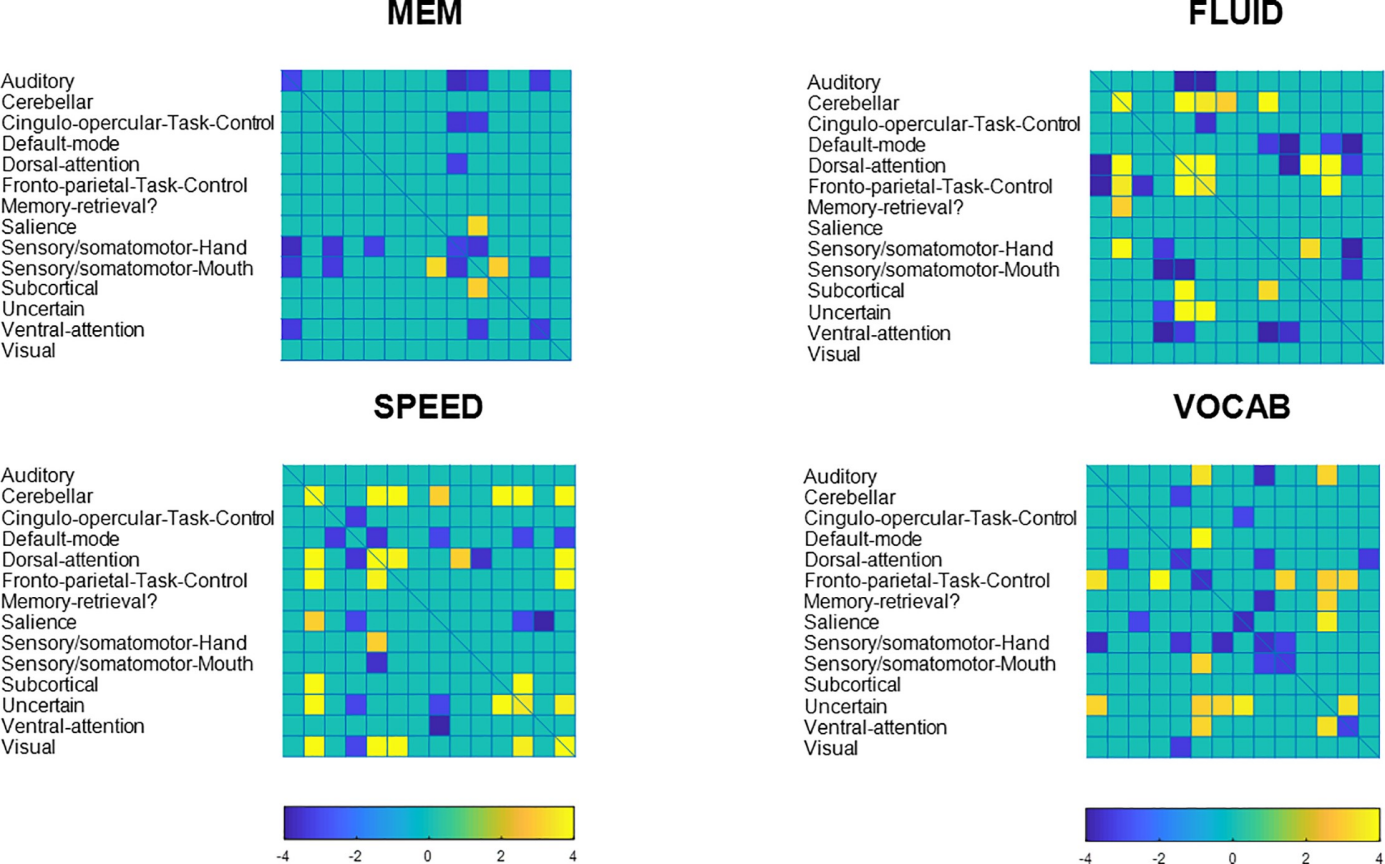
Coarse-grained connectome plot for fluid reasoning. Positive loadings, Z>3, are denoted with warm colors, negative loadings, Z<-3, are denoted with cold colors.

We next turned our attention to the topographic organization of the patterns themselves. The procedure for identifying robust voxels or edges was similar for both modalities. We display the activation and connectivity patterns for all cognitive outcomes below in Figs 4 and 5.

For an exhaustive listing of all super-threshold maxima in the voxel-activation patterns are listed in S1–S3 Tables. (Vocabulary did not yield any super-threshold voxels). Network connectivity loadings with |Z|>3 can be found S4–S7 Tables, with listing of significant loadings for all cognitive outcomes.

The pattern composition shows a few general facts: across cognitive domains, the robustness of activation patterns does seem to track the predictive success out of sample. Fluid Reasoning showed best predictive success, and offered the most robust activation and connectivity patterns, while the converse was true for memory. Negative loadings on inter-network connectivity seem to play an important role, particularly for Memory and Vocabulary.

## 4. Discussion

The current study set out on a narrow empirical comparison of functional connectivity and voxel activation in the same participants and tasks. For cases without clear mechanistic rationales which would dictate the choice of functional connectivity as an outcome, empirical comparisons can map out the differential performance between voxel activation and functional connectivity as inputs for the prediction of behavioral or clinical subject information. Since the mere presence of task-fMRI implies the availability of both functional connectivity and voxel activation (which are just 1^st^ and 2^nd^ order moments of the underlying activation time series), voxel activation is a natural reference standard for prediction performance since it arguably is less confounded by artefacts than functional connectivity.

One caveat is that our survey is contingent on the analytic technique of choice: The Scaled Subprofile Model (a form of PCA regression) which has a long history of successful application in neuroimaging. Since many other techniques are available, we cannot claim that our results would generalize across techniques. However, SSM is a good start for an interrogation since it is a simple and “shallow” multivariate technique, which nevertheless takes advantage of the data in a parsimonious way.

### 4.1 Summary of salient points

We summarize the most salient points from our investigation.

#### 4.1.1 Functional connectomes show larger subject differences than voxel activation

In this report, we have shown several findings: functional connectivity possesses a flatter variance spectrum, hinting at possible greater noise contributions, than voxel activation. This is consistent with the expectation from stable trait effects of connectivity [31], suggesting larger inter-subjective differences in functional connectivity relative to within-subject functional modulations, in contrast to voxel activation.

#### 4.1.2 Predictive utility for brain-behavior relationships shows no clear advantages of functional connectivity over voxel activation

Functional connectivity did not show any clear-cut advantages over voxel activation, and only modest advantages were noted for Memory and Fluid-Reasoning tasks. Any mean differences in the predictive utility of the two modalities are dwarfed by the size of the within-modality variability.

#### 4.1.3 For memory, perceptual speed and vocabulary tasks, brain imaging provides no compelling advantage over a minimal demographic reference model

This was an unexpected, somewhat finding: a reference model using the basic demographics of age, education, and sex was adopted for our comparative survey, to estimate how much extra information functional or structural brain imaging provides in an honest apples-to-apples comparison. For FLUID tasks, functional (but not structural) brain imaging showed clear added predictive utility, but for MEM, SPEED and VOCAB tasks, this was not the case. The 3 readily available demographics used in the reference model ceded barely any advantage in an estimation of cognitive performance to the more sophisticated brain-imaging techniques. For better prediction, significantly more data and possibly more complicated learning architectures need to be employed before the extra effort of brain-data acquisition pays off.

### 4.2 Relationship between voxel activation and functional connectivity in the wider literature

Previous investigations have considered the mechanistic link between activation and connectivity [32, 33]. Local relationships between connectivity and activation are theoretically plausible and activation has been found to be positively correlated with local and global “hubness” properties, particular in areas of the perceptual periphery in occipital and parietal cortex. The task used in [33] was a basic perceptual eye-tracking task, whereas the tasks in [32] were more complicated and used social as well relational demands. Nevertheless, the positive relation between local synchronization and activation was shown for both studies. In contrast, the current report considered voxel activation and functional connectivity strictly in relation to behavioral performance. It was agnostic to questions of local mechanisms. Apriori, a strong task-related coupling of 2 areas does not automatically imply that these areas also significantly active, and vice versa. Whether functional connectivity and voxel activation provide collinear information across participants was therefore an open empirical question, which we assessed by combining the out-of-sample predictions from both modalities through averaging into a “vote”. A vote whose prediction surpasses the prediction of either modality would signal that each modality provided unique information that is useful for the prediction of the cognitive outcome. It also implies that there are irreducible parts which make it impossible to explain the behavior of one modality in terms of the other modality. In our results we found such synergy to a minimal degree. The medians and interquartile ranges of the PRESS statistic were usually lower for the vote than for either activation, connectivity, or brain structure considered in isolation, but the differences were small in relation to the interquartile ranges themselves.

## 5. Conclusion

We conclude our study by repeating the caveat that the results are contingent on one analytic technique, and that other techniques possibly might give diverging answers. We also repeat that the perspective of empirical predictions and relationships to cognitive performance is narrow: clear mechanistic questions centered on the interaction of select regions obviously compel the choice of functional connectivity. Short of such apriori constraints, we can say that functional connectivity shows no clear superiority over activation when it comes to brain-behavioral predictions. The best strategy from the standpoint of prediction might be to combine activation and functional connectivity to derive multimodal predictive patterns for the best predictive utility. The synergy of such an approach might become more pronounced with higher numbers of observations, giving a stronger rationale for the combined approach for large data sets.

## Supporting information

S1 Table Robust loadings for MEM activation pattern at |Z|>3, cluster size >100. (DOCX)

S2 Table Robust loadings for FLUID activation pattern at |Z|>3, cluster size >100. (DOCX)

S3 Table Robust loadings for SPEED activation pattern at |Z|>3, cluster size >100. (DOCX)

S4 Table Robust loadings for coarse-grained MEM connectivity pattern at |Z|>3. (DOCX)

S5 Table Robust loadings for coarse-grained FLUID connectivity pattern at |Z|>3. (DOCX)

S6 Table Robust loadings for coarse-grained SPEED connectivity pattern at |Z|>3. (DOCX)

S7 Table Robust loadings for coarse-grained VOCAB connectivity pattern at |Z|>3. (DOCX)
