# Supplementary material for: Predictive utility of task-related functional connectivity vs. voxel activation
Source: PLoS One. 2021 Apr 8;16(4):e0249947. doi: 10.1371/journal.pone.0249947 (PMC8031148; doi:10.1371/journal.pone.0249947)
Supplement: S1 Table — (DOCX) [file pone.0249947.s001.docx]

| **MNI-X** | **MNI-Y** | **MNI-Z** | **CS** | **Z** |  |
| --- | --- | --- | --- | --- | --- |
| Positive Loadings | | | | | |
| 27 | -75 | -15 | 161 | 3.4892 | Fusiform_R |
| 36 | -60 | -18 | 161 | 3.4568 | Fusiform_R |
| Negative Loadings | | | | | |
| -3 | -21 | 33 | 104 | -3.4276 | Cingulum_Mid_L |
| 6 | 36 | 18 | 249 | -3.3984 | Cingulum_Ant_R |
| -3 | 42 | 6 | 249 | -3.3323 | Cingulum_Ant_L |
| -3 | 21 | 21 | 249 | -3.3256 | Cingulum_Ant_L |
| 9 | -33 | 51 | 104 | -3.3141 | Paracentral_Lobule_R |
| 0 | -33 | 39 | 104 | -3.2434 | Cingulum_Mid_L |
| 6 | -15 | 42 | 104 | -3.2366 | Cingulum_Mid_R |
| 9 | 36 | -6 | 249 | -3.1346 | Cingulum_Ant_R |
| 9 | 36 | 36 | 249 | -3.0972 | Cingulum_Mid_R |

S1 Table: Robust loadings for MEM activation pattern at |Z|>3, cluster size >100.
