# Supplementary material for: Predictive utility of task-related functional connectivity vs. voxel activation
Source: PLoS One. 2021 Apr 8;16(4):e0249947. doi: 10.1371/journal.pone.0249947 (PMC8031148; doi:10.1371/journal.pone.0249947)
Supplement: S2 Table — (DOCX) [file pone.0249947.s002.docx]

S2 Table: Robust loadings for FLUID activation pattern at |Z|>3, cluster size >100.

| **MNI-X** | **MNI-Y** | **MNI-Z** | **CS** | **Z** | **AAL_label** |
| --- | --- | --- | --- | --- | --- |
| Positive Loadings | | | | | |
| 0 | -45 | -21 | 1655 | 8.9794 | Vermis_3 |
| 12 | -54 | -9 | 1655 | 8.074 | Cerebellum_4_5_R |
| -15 | -48 | -15 | 1655 | 7.9503 | Cerebellum_4_5_L |
| 21 | -45 | -24 | 1655 | 7.5305 | Cerebellum_4_5_R |
| 3 | -63 | -15 | 1655 | 7.3323 | Vermis_6 |
| -9 | -66 | -12 | 1655 | 7.2654 | Cerebellum_6_L |
| -24 | -39 | -27 | 1655 | 7.1725 | Cerebellum_4_5_L |
| 24 | -33 | -27 | 1655 | 7.1693 | Cerebellum_4_5_R |
| 21 | -57 | -24 | 1655 | 7.0945 | Cerebellum_6_R |
| 9 | -54 | 33 | 1655 | 7.0646 | Precuneus_R |
| -18 | -60 | -18 | 1655 | 6.9446 | Cerebellum_6_L |
| 54 | -54 | 21 | 237 | 6.8573 | Temporal_Mid_R |
| 15 | -72 | 21 | 1655 | 6.7038 | Cuneus_R |
| -18 | -69 | 15 | 1655 | 6.2882 | Calcarine_L |
| 57 | -21 | -6 | 159 | 6.1943 | Temporal_Mid_R |
| -9 | -60 | 30 | 1655 | 6.0537 | Precuneus_L |
| -54 | -60 | 24 | 237 | 5.6394 | Temporal_Mid_L |
| 54 | -36 | 27 | 159 | 5.6323 | SupraMarginal_R |
| -6 | -48 | -6 | 1655 | 5.5346 | Cerebellum_4_5_L |
| 24 | -60 | 12 | 1655 | 5.5009 | Calcarine_R |
| -3 | -69 | 18 | 1655 | 5.2551 | Calcarine_L |
| 36 | -42 | -27 | 1655 | 5.0645 | Cerebellum_6_R |
| -9 | -78 | 27 | 1655 | 4.9012 | Cuneus_L |
| -45 | -60 | 33 | 237 | 4.7365 | Angular_L |
| -18 | -66 | 0 | 1655 | 4.6647 | Lingual_L |
| 48 | -69 | 42 | 237 | 4.6065 | Angular_R |
| 66 | -30 | 3 | 159 | 4.4018 | Temporal_Sup_R |
| -66 | -36 | 15 | 237 | 4.3655 | Temporal_Sup_L |
| -42 | -18 | 15 | 237 | 4.2741 | Rolandic_Oper_L |
| -54 | -33 | 21 | 237 | 4.2491 | Temporal_Sup_L |
| 54 | -66 | 15 | 237 | 4.1707 | Temporal_Mid_R |
| -39 | -72 | 42 | 237 | 4.0734 | Angular_L |
| 6 | -60 | 12 | 1655 | 3.9455 | Calcarine_R |
| -48 | -45 | 21 | 237 | 3.9119 | Temporal_Sup_L |
| 57 | -57 | 6 | 237 | 3.8767 | Temporal_Mid_R |
| -45 | -63 | 9 | 237 | 3.7931 | Temporal_Mid_L |
| 33 | -72 | -21 | 1655 | 3.7561 | Cerebellum_6_R |
| 60 | -18 | 15 | 159 | 3.6646 | Rolandic_Oper_R |
| 60 | -45 | 9 | 159 | 3.6613 | Temporal_Mid_R |
| 9 | -51 | 48 | 1655 | 3.3458 | Precuneus_R |
| -15 | -33 | -15 | 1655 | 3.2495 | Fusiform_L |
| Negative Loadings | | | | | |
| -27 | -96 | 3 | 256 | -9.316 | Occipital_Mid_L |
| 21 | 6 | 6 | 860 | -8.476 | Putamen_R |
| -24 | -93 | 15 | 256 | -8.275 | Occipital_Mid_L |
| 30 | -87 | 15 | 196 | -7.453 | Occipital_Mid_R |
| 12 | -84 | -9 | 143 | -7.284 | Lingual_R |
| 21 | 9 | -9 | 860 | -7.244 | Putamen_R |
| -24 | -75 | -9 | 105 | -6.879 | Fusiform_L |
| 33 | -72 | 27 | 196 | -6.528 | Occipital_Mid_R |
| -24 | -75 | 42 | 256 | -6.306 | Occipital_Sup_L |
| -18 | 9 | 0 | 860 | -6.236 | Putamen_L |
| 9 | -6 | 6 | 860 | -5.983 | Thalamus_R |
| -27 | -81 | 21 | 256 | -5.974 | Occipital_Mid_L |
| -33 | 15 | 3 | 860 | -5.953 | Insula_L |
| 21 | 36 | -15 | 860 | -5.688 | Frontal_Sup_Orb_R |
| 27 | -75 | 45 | 196 | -5.642 | Occipital_Sup_R |
| -9 | -99 | 0 | 105 | -5.258 | Calcarine_L |
| 36 | 15 | 0 | 860 | -5.2 | Insula_R |
| -18 | -69 | 51 | 256 | -5.163 | Parietal_Sup_L |
| 33 | -90 | -3 | 196 | -5.127 | Occipital_Inf_R |
| 24 | 24 | -18 | 860 | -5.123 | Frontal_Inf_Orb_R |
| -12 | -84 | -9 | 105 | -4.969 | Lingual_L |
| 27 | -63 | -6 | 143 | -4.929 | Fusiform_R |
| -30 | -63 | -12 | 105 | -4.907 | Fusiform_L |
| 15 | -93 | 18 | 143 | -4.853 | Occipital_Sup_R |
| -36 | -84 | 12 | 256 | -4.846 | Occipital_Mid_L |
| 0 | 9 | 51 | 121 | -4.82 | Supp_Motor_Area_L |
| -9 | 6 | 12 | 860 | -4.747 | Caudate_L |
| -6 | 24 | 42 | 121 | -4.67 | Frontal_Sup_Medial_L |
| 15 | -93 | 3 | 143 | -4.668 | Calcarine_R |
| -12 | -15 | 3 | 860 | -4.61 | Thalamus_L |
| 27 | -63 | 48 | 196 | -4.597 | Angular_R |
| -30 | -57 | 54 | 256 | -4.54 | Parietal_Inf_L |
| -30 | -51 | 42 | 256 | -4.525 | Parietal_Inf_L |
| -9 | 12 | 33 | 121 | -4.416 | Cingulum_Mid_L |
| -6 | 33 | 33 | 121 | -4.22 | Frontal_Sup_Medial_L |
| 39 | 18 | -27 | 860 | -4.142 | Temporal_Pole_Sup_R |
| 30 | 9 | -27 | 860 | -4.081 | Temporal_Pole_Sup_R |
| 36 | -81 | 6 | 196 | -3.978 | Occipital_Mid_R |
| -9 | 21 | -3 | 860 | -3.868 | Caudate_L |
| 42 | 12 | -39 | 860 | -3.724 | Temporal_Pole_Mid_R |
| 9 | 12 | 36 | 121 | -3.38 | Cingulum_Mid_R |
| 15 | -75 | 48 | 196 | -3.372 | Precuneus_R |
