# Supplementary material for: Predictive utility of task-related functional connectivity vs. voxel activation
Source: PLoS One. 2021 Apr 8;16(4):e0249947. doi: 10.1371/journal.pone.0249947 (PMC8031148; doi:10.1371/journal.pone.0249947)
Supplement: S3 Table — (DOCX) [file pone.0249947.s003.docx]

S3 Table: Robust loadings for SPEED activation pattern at |Z|>3, cluster size >100.

| **MNI-X** | **MNI-Y** | **MNI-Z** | **CS** | **Z** | **AAL_label** |
| --- | --- | --- | --- | --- | --- |
| Positive Loadings | | | | | |
| 0 | -69 | -18 | 1406 | 9.5204 | Vermis_6 |
| 36 | -42 | -24 | 1406 | 8.8548 | Fusiform_R |
| 24 | -48 | -18 | 1406 | 8.7379 | Cerebellum_4_5_R |
| -24 | -57 | -15 | 1406 | 8.6606 | Fusiform_L |
| 27 | -51 | -30 | 1406 | 8.3903 | Cerebellum_6_R |
| 36 | -66 | -15 | 1406 | 8.0466 | Fusiform_R |
| -30 | -51 | -27 | 1406 | 7.9447 | Cerebellum_6_L |
| 0 | -51 | -21 | 1406 | 7.5602 | Vermis_4_5 |
| 24 | -75 | -9 | 1406 | 7.4555 | Fusiform_R |
| -21 | -72 | -15 | 1406 | 7.3934 | Cerebellum_6_L |
| -9 | -75 | -12 | 1406 | 7.2366 | Cerebellum_6_L |
| 3 | -54 | -3 | 1406 | 6.9127 | Vermis_4_5 |
| 45 | -75 | 0 | 119 | 6.4514 | Occipital_Inf_R |
| 27 | -81 | 30 | 119 | 6.2148 | Occipital_Sup_R |
| 6 | -81 | -15 | 1406 | 6.1397 | Vermis_6 |
| -39 | -66 | -18 | 1406 | 6.1071 | Cerebellum_6_L |
| 12 | -72 | -9 | 1406 | 6.0772 | Lingual_R |
| -21 | -84 | 30 | 100 | 5.794 | Occipital_Sup_L |
| -15 | -63 | 60 | 100 | 5.4362 | Precuneus_L |
| -12 | -33 | -3 | 118 | 5.3029 | Lingual_L |
| 42 | -72 | 12 | 119 | 5.2354 | Occipital_Mid_R |
| 45 | -63 | 0 | 119 | 5.1103 | Temporal_Mid_R |
| -9 | -78 | 48 | 100 | 5.0028 | Precuneus_L |
| 39 | -63 | -27 | 1406 | 4.943 | Cerebellum_Crus1_R |
| -39 | -45 | -18 | 1406 | 4.5908 | Fusiform_L |
| 45 | -75 | -15 | 119 | 4.408 | Occipital_Inf_R |
| -12 | -33 | -24 | 118 | 4.3553 | Cerebellum_3_L |
| 6 | -66 | 60 | 100 | 4.3386 | Precuneus_R |
| 15 | -78 | 45 | 119 | 3.8552 | Cuneus_R |
| 33 | -81 | 15 | 119 | 3.832 | Occipital_Mid_R |
| 36 | -84 | -9 | 119 | 3.8313 | Occipital_Inf_R |
| -27 | -84 | 18 | 100 | 3.2403 | Occipital_Mid_L |
| Negative Loadings | | | | | |
| -39 | 3 | 12 | 174 | -9.9202 | Insula_L |
| -18 | 21 | 57 | 647 | -8.1534 | Frontal_Sup_L |
| 12 | 36 | 54 | 647 | -8.1177 | Frontal_Sup_Medial_R |
| 33 | 24 | 42 | 647 | -8.0393 | Frontal_Mid_R |
| 6 | 24 | 24 | 354 | -7.7949 | Cingulum_Ant_R |
| -9 | 39 | 51 | 647 | -7.6272 | Frontal_Sup_Medial_L |
| -27 | 24 | 39 | 647 | -7.2888 | Frontal_Mid_L |
| -48 | -6 | 12 | 174 | -6.8357 | Rolandic_Oper_L |
| -48 | 24 | 9 | 174 | -6.7704 | Frontal_Inf_Tri_L |
| -3 | 18 | 30 | 354 | -6.7619 | Cingulum_Ant_L |
| 18 | 27 | 60 | 647 | -6.6573 | Frontal_Sup_R |
| -39 | 15 | 54 | 647 | -6.2693 | Frontal_Mid_L |
| -18 | 39 | 42 | 647 | -6.2653 | Frontal_Sup_L |
| -42 | 30 | 0 | 174 | -5.9956 | Frontal_Inf_Tri_L |
| 0 | 39 | 18 | 354 | -5.8891 | Cingulum_Ant_L |
| 39 | 15 | 51 | 647 | -5.8713 | Frontal_Mid_R |
| -9 | 45 | 12 | 354 | -5.8249 | Cingulum_Ant_L |
| 21 | 30 | 45 | 647 | -5.5858 | Frontal_Mid_R |
| -45 | 15 | 39 | 647 | -5.5568 | Frontal_Mid_L |
| 12 | 45 | 6 | 354 | -5.2377 | Cingulum_Ant_R |
| -9 | 24 | 39 | 354 | -3.7896 | Cingulum_Mid_L |
