# Supplementary material for: Predictive utility of task-related functional connectivity vs. voxel activation
Source: PLoS One. 2021 Apr 8;16(4):e0249947. doi: 10.1371/journal.pone.0249947 (PMC8031148; doi:10.1371/journal.pone.0249947)
Supplement: S4 Table — (DOCX) [file pone.0249947.s004.docx]

S4 Table: Robust loadings for coarse-grained MEM connectivity pattern at |Z|>3.

| **Network1** | **Network2** | **Z** |
| --- | --- | --- |
| **Positive Loadings** | | |
| Salience | Sensory/somatomotor_Mouth | 3.2919 |
| Sensory/somatomotor_Mouth | Subcortical | 3.0062 |
| **Negative Loadings** | |  |
| Auditory | Sensory/somatomotor_Hand | -3.6277 |
| Cingulo-opercular_Task_Control | Sensory/somatomotor_Hand | -3.4954 |
| Sensory/somatomotor_Hand | Sensory/somatomotor_Mouth | -3.3966 |
| Auditory | Sensory/somatomotor_Mouth | -3.3818 |
| Cingulo-opercular_Task_Control | Sensory/somatomotor_Mouth | -3.3721 |
| Sensory/somatomotor_Mouth | Ventral_attention | -3.3459 |
| Auditory | Ventral_attention | -3.3195 |
| Ventral_attention | Ventral_attention | -3.276 |
| Sensory/somatomotor_Hand | Sensory/somatomotor_Hand | -3.1655 |
| Dorsal_attention | Sensory/somatomotor_Hand | -3.1315 |
| Auditory | Auditory | -3.0025 |
