# Supplementary material for: Predictive utility of task-related functional connectivity vs. voxel activation
Source: PLoS One. 2021 Apr 8;16(4):e0249947. doi: 10.1371/journal.pone.0249947 (PMC8031148; doi:10.1371/journal.pone.0249947)
Supplement: S5 Table — (DOCX) [file pone.0249947.s005.docx]

S5 Table: Robust loadings for coarse-grained FLUID connectivity pattern at |Z|>3.

| **Network1** | **Network2** | **Z** |
| --- | --- | --- |
| **Positive Loadings** | | |
| Dorsal_attention | Fronto-parietal_Task_Control | 7.5967 |
| Dorsal_attention | Dorsal_attention | 6.53 |
| Dorsal_attention | Uncertain | 5.9452 |
| Cerebellar | Sensory/somatomotor_Hand | 4.8089 |
| Cerebellar | Cerebellar | 4.6136 |
| Dorsal_attention | Subcortical | 4.5002 |
| Fronto-parietal_Task_Control | Uncertain | 4.219 |
| Cerebellar | Dorsal_attention | 4.0418 |
| Fronto-parietal_Task_Control | Fronto-parietal_Task_Control | 3.6064 |
| Cerebellar | Fronto-parietal_Task_Control | 3.5183 |
| Sensory/somatomotor_Hand | Subcortical | 3.334 |
| Cerebellar | Memory_retrieval? | 3.0981 |
| **Negative Loadings** | | |
| Auditory | Dorsal_attention | -7.2635 |
| Default_mode | Sensory/somatomotor_Mouth | -4.2748 |
| Sensory/somatomotor_Hand | Ventral_attention | -4.1782 |
| Auditory | Fronto-parietal_Task_Control | -4.1408 |
| Dorsal_attention | Sensory/somatomotor_Mouth | -3.9605 |
| Default_mode | Ventral_attention | -3.9229 |
| Sensory/somatomotor_Mouth | Ventral_attention | -3.5763 |
| Cingulo-opercular_Task_Control | Fronto-parietal_Task_Control | -3.5499 |
| Dorsal_attention | Ventral_attention | -3.3352 |
| Default_mode | Sensory/somatomotor_Hand | -3.3248 |
| Default_mode | Uncertain | -3.1074 |
