# Supplementary material for: Predictive utility of task-related functional connectivity vs. voxel activation
Source: PLoS One. 2021 Apr 8;16(4):e0249947. doi: 10.1371/journal.pone.0249947 (PMC8031148; doi:10.1371/journal.pone.0249947)
Supplement: S6 Table — (DOCX) [file pone.0249947.s006.docx]

S6 Table: Robust loadings for coarse-grained SPEED connectivity pattern at |Z|>3.

| **Network 1** | **Network 2** | **Z** |
| --- | --- | --- |
| **Positive Loadings** | | |
| Cerebellar | Visual | 6.2348 |
| Cerebellar | Dorsal_attention | 6.128 |
| Dorsal_attention | Visual | 6.1136 |
| Cerebellar | Uncertain | 5.8641 |
| Dorsal_attention | Dorsal_attention | 5.6183 |
| Cerebellar | Cerebellar | 5.4723 |
| Visual | Visual | 5.3227 |
| Fronto-parietal_Task_Control | Visual | 4.8221 |
| Cerebellar | Subcortical | 4.4512 |
| Cerebellar | Fronto-parietal_Task_Control | 4.2229 |
| Subcortical | Uncertain | 3.9959 |
| Dorsal_attention | Fronto-parietal_Task_Control | 3.8138 |
| Uncertain | Visual | 3.6403 |
| Uncertain | Uncertain | 3.5583 |
| Dorsal_attention | Sensory/somatomotor_Hand | 3.0701 |
| Cerebellar | Salience | 3.0001 |
| **Negative Loadings** | | |
| Salience | Ventral_attention | -3.925 |
| Dorsal_attention | Sensory/somatomotor_Mouth | -3.5897 |
| Default_mode | Dorsal_attention | -3.3859 |
| Cingulo-opercular_Task_Control | Default_mode | -3.3414 |
| Salience | Uncertain | -3.0876 |
| Default_mode | Uncertain | -3.0652 |
| Default_mode | Salience | -3.0394 |
| Default_mode | Visual | -3.0176 |
