# Supplementary material for: Predictive utility of task-related functional connectivity vs. voxel activation
Source: PLoS One. 2021 Apr 8;16(4):e0249947. doi: 10.1371/journal.pone.0249947 (PMC8031148; doi:10.1371/journal.pone.0249947)
Supplement: S7 Table — (DOCX) [file pone.0249947.s007.docx]

S7 Table: Robust loadings for coarse-grained VOCAB connectivity pattern at |Z|>3.

| **Network 1** | **Network 2** | **Z** |
| --- | --- | --- |
| **Positive Loadings** | | |
| Default_mode | Fronto-parietal_Task_Control | 3.9049 |
| Salience | Uncertain | 3.676 |
| Auditory | Fronto-parietal_Task_Control | 3.4965 |
| Uncertain | Ventral_attention | 3.4141 |
| Memory_retrieval? | Uncertain | 3.1945 |
| Fronto-parietal_Task_Control | Ventral_attention | 3.1744 |
| Fronto-parietal_Task_Control | Sensory/somatomotor_Mouth | 3.1724 |
| Auditory | Uncertain | 3.151 |
| Fronto-parietal_Task_Control | Uncertain | 3.0122 |
| **Negative Loadings** | | |
| Auditory | Sensory/somatomotor_Hand | -3.7462 |
| Memory_retrieval? | Sensory/somatomotor_Hand | -3.7225 |
| Salience | Salience | -3.6272 |
| Sensory/somatomotor_Hand | Sensory/somatomotor_Hand | -3.5974 |
| Fronto-parietal_Task_Control | Fronto-parietal_Task_Control | -3.5569 |
| Dorsal_attention | Dorsal_attention | -3.4695 |
| Dorsal_attention | Sensory/somatomotor_Hand | -3.4149 |
| Dorsal_attention | Visual | -3.3119 |
| Sensory/somatomotor_Mouth | Sensory/somatomotor_Mouth | -3.2775 |
| Sensory/somatomotor_Hand | Sensory/somatomotor_Mouth | -3.2412 |
| Cerebellar | Dorsal_attention | -3.1883 |
| Ventral_attention | Ventral_attention | -3.1555 |
| Cingulo-opercular_Task_Control | Salience | -3.0283 |
